# The Effect of High-Altitude Acclimatisation on Ultra-Short Heart Rate Variability

**DOI:** 10.3389/fcvm.2022.787147

**Published:** 2022-03-28

**Authors:** Christopher John Boos, Adrian Mellor, David Richard Woods, John Paul O’Hara

**Affiliations:** ^1^Department of Cardiology, Poole Hospital, University Hospitals Dorset, Poole, United Kingdom; ^2^Department of Postgraduate Medical Education, Bournemouth University, Bournemouth, United Kingdom; ^3^Carnegie School of Sport, Leeds Beckett University, Leeds, United Kingdom; ^4^Defence Medical Services, Lichfield, United Kingdom; ^5^James Cook University Hospital, Middlesbrough, United Kingdom; ^6^Northumbria NHS Foundation Trust, North Shields, United Kingdom; ^7^Academic Department of Medicine, University of Newcastle, Newcastle upon Tyne, United Kingdom

**Keywords:** high-altitude, heart rate variability, acute mountain sickness, oxygen saturation, acclimatisation

## Abstract

**Introduction:**

High-altitude (HA) exposure affects heart rate variability (HRV) and has been inconsistently linked to acute mountain sickness (AMS). The influence of increasing HA exposure on ultra-short HRV and its relationship to gold standard HRV measures at HA has not been examined.

**Methods:**

This was a prospective observational study of adults aged ≥ 18 years undertaking a HA trek in the Dhaulagiri region of the Himalayas. Cardiac inter-beat-intervals were obtained from a 10-s recording of supra-systolic blood pressure (Uscom BP^+^ device) immediately followed by 300 s single lead ECG recording (CheckMyHeart device). HRV was measured using the RMSSD (root mean square of successive differences of NN intervals) at sea level (SL) in the United Kingdom and at 3,619, 4,600, and 5,140 m at HA. Oxygen saturations (SpO_2_) were measured using finger-based pulse oximetry. The level of agreement between the 10 and 300 s RMSSD values were examined using a modified Bland–Altman relative-difference analysis.

**Results:**

Overall, 89 participants aged 32.2 ± 8.8 years (range 18–56) were included of which 70.8% were men. HA exposure (SL vs. 3,619 m) was associated with an initial increase in both 10 s (45.0 [31.0–82.0]) vs. 58.0 [33.0–119.0] ms) and 300 s (45.67 [33.24–70.32] vs. 56.48 [36.98–102.0] ms) in RMSSD. Thereafter at 4,600 and 5,140 m both 10 and 300 s RMSSD values were significantly lower than SL. From a total of 317 paired HRV measures the 10 and 300 s RMSSD measures were moderately correlated (Spearman *r* = 0.66; 95% CI: 0.59–0.72; *p* < 0.0001). The median difference (bias) in RMSSD values (300 s − 10 s) was −2.3 ms with a lower and upper limit of agreement of −107.5 and 88.61 ms, respectively with no differences with altitude. Overall, 293/317 (92.4%) of all paired HRV values fell within the 95% CI limits of agreement. Neither HRV method was predictive of AMS.

**Conclusion:**

Increasing HA affects ultra-short HRV in a similar manner to gold-standard 300 s. Ultra-short HRV has a moderate agreement with 300 s measurements. HRV did not predict AMS.

## Introduction

The physiological responses to the high-altitude (HA) environment are unpredictable and highly individualised, depending on several factors, such as age, sex, genetics, background fitness, perceived exertion, and mood (1, 2). They are further influenced by a multitude of external factors including the ascent profile, altitude gain and exercise burden (1). Increasing hypobaric hypoxia leads to sympathetic activation and an increase in resting heart rate. Conversely, competing parasympathetic activation contributes to blunting of the maximal heart rate, cardiac output, and peak oxygen consumption.

One of the greatest challenges in HA research is the difficulties in the early identification of individuals who can be potentially harmed by the consequences of significant HA-related symptoms and acute mountain sickness (AMS). AMS is known to affect more than 25% of persons ascending to >2,500 m above sea level (SL), with an incidence that is heavily influenced by the speed and mode of ascent, HA environment and altitude gained (1, 3).

Quantification of the variance in cardiac inter-beat intervals, known as heart rate variability (HRV) has emerged from being exclusively a research tool to an increasingly useful measure of cardiovascular fitness and physical readiness (4). It provides a non-invasive measure of autonomic balance. Recent technological advances have led to increased portability of HRV measurement tools and growing interest in the ability to provide a useful measure of HRV over shorter time frames than the traditional 5-min recording of heart rate (5). This has created wide ranging research opportunities for HRV research at HA (6). It has been shown that increasing HA is associated with significant alterations in HRV (7, 8). The observed changes appear to be heavily influenced by the ascent profile and individual acclimatisation and its relationship to AMS remains uncertain (6–11).

Ultra-short HRV acquisition using inter-beat recording of under 5 min has gained recent research and clinical traction with enhanced opportunities for HA research. For example, the 55 s finger-sensor derived HRV measure using lnRMSSD (root mean square of successive differences of NN intervals) provided by the ithlete™ device (HRV Fit Ltd., Southampton, United Kingdom) has been shown to strongly agree with gold-standard 300 s (5-min) ECG recording of HRV (12). One major challenge with this device is the ability to provide an accurate and reproducible pulse waveform signal using its finger sensor at HA (12). The Uscom BP^+^ is unique in that it provides a validated RMSSD HRV measurement [known as pulse rate variability (PRV)] from a 10 s recording of the pulse rate using a brachial blood pressure cuff inflated to supra-systolic pressure (13). Its accuracy and agreement with gold standard 5-min HRV has not been investigated.

The aims of this study were twofold: (1) to compare HRV using a brachial-based analysis of the supra-systolic pulse wave over 10 s with the gold standard 5-min recording period and (2) to evaluate the potential association between HRV and AMS using these two methods? We hypothesised that a strong agreement ultra-short HRV and standard HRV measure would persist at HA.

## Materials and Methods

### Study Design and Participants

This was a prospective observational cohort study of adult British Military servicemen who undertook a progressive HA ascent in the Dhaulagiri region of the Himalayas in April/May of 2016. The detailed study design and protocol paper has been previously published (14). All subjects were known low altitude dwellers, aged ≥18 years and were declared medically fit for HA exposure by their medical practitioners to be included. Health status was confirmed following a detailed baseline questionnaire. Persons with a history of cardiac arrhythmias were excluded. The participants were assessed fully rested at SL (<200 m) and at 3,619, 4,600, and 5,140 m at HA. The baseline SL assessments were performed in the United Kingdom at approximately 6 weeks prior to departure.

### High-Altitude Ascent and Descent Profile

The participants flew from the United Kingdom to Kathmandu (1,400 m) where they underwent 2 days of local acclimatisation (days 1–3), after which they travelled by road over 2 days to 1,030 m (Darbang) to start their trekking ascent. From there they trekked on foot to sleeping altitudes of 3,619 m (days 6–7), 4,600 m (day 9), and 5,140 m (day 11, with an overpass of 5,360 m) before commencing their decent on foot to Marpha (2,719 m) and then by road back to Kathmandu. Research assessments were performed at SL and at static research camps at 3,619, 4,600, and 5,140 m during HA ascent.

### Physiological Assessments and Heart Rate Variability

All subjects were rested for at least 5 min prior to their physiological assessment and they were not permitted to drink caffeine or smoke for at least 3 h and alcohol for ≥10 h prior (14). Measurements were taken on fully rested subjects in the early morning post-micturition after sleeping at the designated HA and prior to breakfast or caffeine consumption. Oxygen saturations (SpO_2_) were measured using a Nonin Onyx (Nonin Medical Inc., Plymouth, MN, United States) pulse oximeter with sampling over approximately 15 s. Blood pressure, heart rate and ultra-short HRV were measured using an Uscom BP^+^ device (Uscom, Sydney, NSW, Australia) as previously reported and using brachial blood pressure cuff placed on the dominant arm of seated subjects (15, 16). Following an initial inflation-deflation the cuff was re-inflated to approximately ≥30 mmHg above the recorded systolic blood pressure and to supra-systolic pressure for 10 s, during which supra-systolic BP and pulse wave assessments were recorded *via* the arm cuff. The BP^+^ HRV is calculated as the RMSSD of the detected pulse periods during the recording of the supra-systolic pulse pressure wave using the foot-to-foot interval of each BP pulse at a sampling rate 250 Hz. All recordings were stored on a mini-SD card within the device and later exported for data analysis and full image disclosure of the rhythm trip. Only readings with a signal-to-noise ratio of ≥6 were included and tests with a ratio of <6 were repeated or excluded where a satisfactory signal could not be obtained (9).

A 300 s examination of HRV was conducted immediately after the measurement of blood pressure, heart rate and the ultra-short Uscom BP^+^ HRV recordings. This was performed using battery-operated portable HRV devices [CheckMyHeart (CMH) Plus™ R30 V4, Daily Care Biomedical, Taiwan] as previously described (9, 12). This device records a single lead ECG at a sampling rate of 250 Hz using two surface ECG electrodes placed at the right sternal edge and cardiac apex respectively (9, 12). All physiological measurements were conducted in a temperature-controlled room at SL and whilst wearing warm clothing and in a tent at HA. All stored recordings were exported *via* USB hook up for offline data analysis.

The R waves of the stored ECG were used as the fiducial point to determine the beat-to-beat interval with full ECG disclosure. The RMSSD was calculated from this 300 s recording. HA-related symptoms were recorded using the Lake Louis Scoring (LLS) system (17). AMS was defined as a total lake Louise Score of ≥3 in the presence of headache and recent altitude gain (18).

### Ethics

Participation was entirely voluntary, and all subjects underwent detailed written informed consent prior to inclusion. This study was approved by the Ministry of Defence Research and Medical Ethics Committee (MoDREC) and was conducted according to the standards of the Declaration of Helsinki.

### Statistical Analysis

Data were analysed using GraphPad Prism version 6.07 for Windows (GraphPad Software, San Diego, CA, United States). Sample size calculations were performed using a proprietary determined sample-size calculator using GraphPad StatMate version 2.00 for Windows. Data inspection and the D’Agostino-Pearson Test was undertaken to assess normality of all continuous data, which were presented as mean ± standard deviation (SD) and median [interquartile range] for highly skewed data, respectively.

Categorical variables were compared using Chi-squared tests. Comparison of 10 and 300 s RMSSD values were compared using the Wilcoxon matched-pairs signed-ranks test. Comparisons of continuous data over the four altitudes were performed using a one wave ANOVA and Tukey post-tests and the Kruskal–Wallis Test (non-parametric ANOVA) with the Dunn post-test as appropriate. Correlations were performed using Pearson and Spearman rank correlation (±95% confidence interval, CI) as appropriate.

The association between BP^+^ 10 s and the CMH 300 s RMSSD HRV measures were examined using correlations. Their level of agreement was investigated using a modified Bland–Altman relative-difference plot method as previously described (19–21). This was performed as the 10 and 300 s RMSSD values and their differences were highly skewed and not normally distributed. The differences between the 300 and 10 s RMSSD measures (*y*-axis) were compared with the average values from the comparative readings (*x*-axis). The 95% limits of agreement were estimated using the 2.5th and 97.5th percentile of the differences (300 s − 10 s) in RMSSD values and the average bias estimated as the median of the differences. A good agreement between the comparative RMSSD was defined as ≥95% of readings falling within these limits (21). The differences between the 300 and 10 s were further examined by altitude. Binary Logistic regression [odds ratio (OR) and 95% CI] was used to examine the relationship between AMS (yes/no) and HRV.

A two-tailed *p*-value < 0.05 was considered statistically significant for all comparisons.

### Sample Size Calculations

In a previous observational study Huang et al. reported a significant change in spectral measures of HRV among 32 volunteers who travelled to 3,440 from 1,317 m over a 12 days period (7). In another study our group observed a significant change in lnRMSSD from near SL and HA at >3,600 m in 22 volunteers using a 55 s finger-based recording of HRV (12). Anticipating greater variability with a 10 s HRV recording we calculated that a sample size at least doubling these previous studies would provide sufficient power to detect a significant change in HRV from SL to HA at >3,600 m.

## Results

Overall, 89 participants aged 18–56 years were included of which 70.8% were men. The participant demographics are shown in Table 1. HA was associated with a significant increase in resting heart rate (recorded with BP^+^ and CMH devices), systolic blood pressure and fall in SpO_2_ compared with baseline SL (Table 2). The prevalence of AMS increased from 10.7% at 3,619 m to >20% at both 4,600 and 5,140 m (Table 2). Overall, 36.7% of the participants had AMS at ≥3,619 m.

**TABLE 1:** Sea level baseline demographics.

Demographic	Result
Number	89
Men	63
Age, years (range)	32.18 ± 8.77 (18–56)
Height, m	1.74 ± 0.09
Weight, kg	73.66 ± 12.20
Body mass index, kg/m^2^	24.29 ± 2.69
Systolic blood pressure	132.84 ± 13.94
Diastolic blood pressure	80.51 ± 14.59
Basic fitness time (minutes) (for a 1.5 mile run)	10:01 ± 1:15
**Ethnicity, %**	
Caucasian	77 (86.5%)
Non-Caucasian	12 (13.5%)
**Smoking status (%)**	
Current	64 (71.9%)
Ex	8 (9.0%)
Never	12 (13.5%)

**TABLE 2:** The effect of high-altitude on physiological variables and heart rate variability.

	Sea level	3,619 m	4,600 m	5,140 m	*p*-Value
Acute mountain sickness, %	0	8/75 (10.7%)	15/74 (20.3%)	15/69 (21.7%)	<0.0001
SpO_2_, %	97.78 ± 1.39	91.85 ± 3.44	82.71 ± 6.34	80.11 ± 5.35	<0.0001^abc^
Systolic blood pressure, mmHg	132.84 ± 13.94	136.90 ± 13.38	138.80 ± 13.31	138.6 ± 13.87	0.017^bc^
**Heart rate/minute**					
CMH 300 s	65.43 ± 12.33	67.27 ± 10.78	74.27 ± 14.16	76.95 ± 13.21	<0.0001^bc^
BP^+^ 10 s	65.25 ± 12.87	69.61 ± 11.76	77.32 ± 15.35	78.18 ± 13.62	<0.0001^abc^
Differences (300 s–10 s)	0.18 ± 11.41	−2.33 ± 8.24	−3.10 ± 7.24	−1.00 ± 8.270	0.10
**HRV RMSSD**					
CMH 300 s	45.67 [33.24–70.32]	56.48 [36.98–102.0]	44.71 [26.51–71.71]	40.92 [22.81–72.58]	0.016^e^
BP^+^ 10 s	45.0 [31.0–82.0]	58.0 [33.0–119.0]	44.0 [23.5–76.5]	36.5 [21.0–76.75)	0.028^e^
Differences (300 s–10 s)	−3.0 (−21.0 to 9.0)	−4.5 (−23.25 to 10.25)	−1.5 (23.5 to 9.25)	−1.0 (−17.50 to 12.50)	0.90

*SL, sea level; SpO_2;_ pulse oximetry-derived oxygen saturation; RMSSD, root mean square of successive differences; CMH, CheckMyHeart 300 s HRV; BP^+^, Uscom 10 s HRV device.*

*p-Value for results of overall group post hoc tests: ^a^SL vs. 3,619 m, ^b^SL vs. 4,600 m, ^c^SL vs. 5,140 m, ^d^3,619 vs. 4,600 m, and ^e^3,619 vs. 5,140 m.*

At HA there were 87, 77, and 78 available 10 s BP^+^ HRV recordings at 3,619, 4,600, and 5,140 m, respectively. The equivalent number of valid CMH 300 s recordings were 87, 79, and 81, respectively. Ten second HRV correlated with SpO_2_ (0.21; 95% CI: 0.07–0.34; *p* = 0.003). There was an inverse correlation between 10 s HRV and age (*r* = −0.34; −0.52 to −0.12; *p* = 0.002), physical fitness (based on 1.5 mile run time, *r* = −0.20; −0.31 to −0.08; *p* = 0.004), and resting 10 s mean heart rate (*r* = −0.39; −0.49 to −0.27; *p* < 0.0001).

**FIGURE 1:**
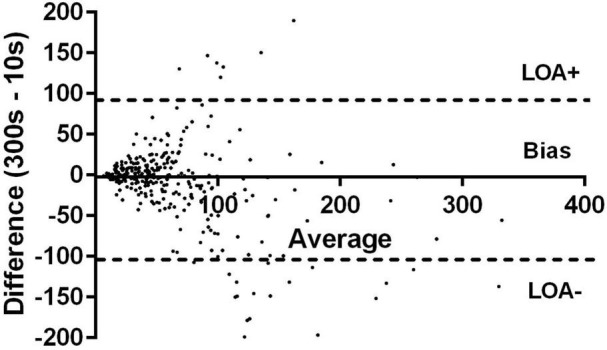
Modified Bland–Altman relative-differences analysis comparing 10 vs. 300 s RMSSD value showing median differences in 10 vs. 300 s and the limits of agreements (LOA) and their 95% confidence intervals.

**FIGURE 2:**
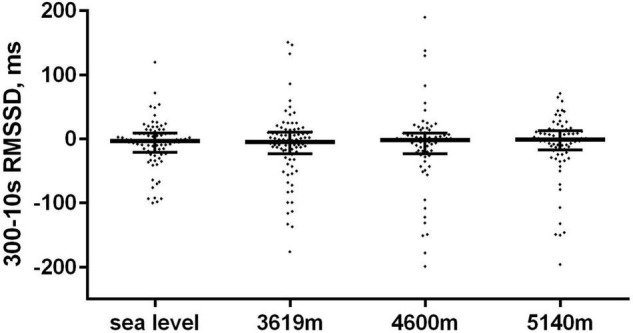
The differences in paired RMSSD (300 s – 10 s) values with increasing high-altitude.

There were 317 paired (10 vs. 300 s) data. There was a moderate correlation between mean 300 and 10 s heart rate (*r* = 0.77: 95% CI: 0.72–0.82; *p* < 0.0001) with no significant paired difference in values (Table 2). HRV measures were moderately correlated (Spearman *r* = 0.66; 95% CI: 0.59–0.72; *p* < 0.0001). The median difference (bias) in RMSSD values between the 300 and 10 s HRV method was −2.3 ms (*p* = 0.005) with a lower and upper limit of agreement (95% CI) of −107.5 and 88.61 ms, respectively (Figure 1). There were no differences in agreement between the 300 and 10 s RMSSD with altitude (Table 2 and Figure 2). For the comparative RMSSD results there were 7 values above the upper limit of agreement and 17 values below; 293/317 (91.4%) of all paired HRV values fell within the 95% CI limits of agreement (Figure 1). There was no significant association between 10 s HRV and AMS (OR 1.002; 95% CI: 0.98–1.007; *p* = 0.56) or with the standard 300 s HRV (OR 1.01; 95% CI: 1.0–1.03; *p* = 0.08) measurements.

## Discussion

This is one of the largest studies to examine HRV at HA and the first to investigate 10 s brachial-based HRV at HA. It is also, to our knowledge, the first to examine its agreement with standard 5-min (300 s) ECG-derived HRV recordings. There was only modest agreement, at best, between 10 and 300 s heart rate and RMSSD, with a tendency to significantly higher RMSSD scores with the 10 s HRV measurement. HRV was not predictive of AMS.

Increasing hypobaric hypoxia with HA ascent is a potent sympathetic activator and leads to a rise in resting heart rate, cardiac output, blood pressure, minute ventilation and pulmonary vascular resistance (22). Paradoxically, the parasympathetic system is also highly activated leading to increased urine output, reduced maximal heart rate and nocturnal bradycardia (22). In this study we examined RMSSD, which is perhaps the most widely reported HRV variable. It is one of the easiest HRV parameters to measure and hence does not require overly complex equipment and has the relative advantage of frequency-domain measures of requiring as little as 10 s of recording time to provide a reading (5, 23). RMSSD is also less prone to the effects of respiration, which is advantageous at HA environment, where hyperventilation predominates (1, 5). It has been shown to be a useful and indirect measure of training adaptation, fitness, and parasympathetic tone which are features of normal HA acclimatisation (22, 24, 25). Conversely, a reduction in RMSSD has been linked to overtraining and fatigue (26). Hence, the measurement of RMSSD HRV is a potentially useful tool to track physiological adaptation to the HA environment. There is data to support an association between HRV and AMS development (7, 8, 10, 27), however, overall the results inconsistent and warrant further research particularly at very HA to >5,000 m where there is very limited previous data (9, 11, 28).

The utility of ultra-short HRV and PRV to replicate gold standard HRV measures has been well investigated (23, 29–32). The agreement has been generally strong when limited to RMSSD HRV data, although the impact of single false heartbeat is considerably greater with ultra-short recording HRV methods. The comparative validation studies were all conducted at SL and using photoplethysmography (PPG) sensors to measure PRV (29). In the only direct comparison at HA, our group compared the agreement in lnRMSSD values between a 55 s (using a finger-based PPG, ithlete™) and 300 s (CheckMyHeart™) recording period among 22 adults undertaking a similar HA ascent profile to our current study (12). We found a far stronger and narrower limits of agreement between methods than in this current study; although again there was tendency to higher RMSSD values with shorter 55 vs. 300 s HRV recording (mean difference 1.8l; 95% CI: −12.3 to 8.5).

This study is the first, to our knowledge, to examine the agreement between 10 s brachial artery derived HRV and 300 s ECG derived HRV measurements. The ability to obtain accurate and reproducible HRV data from such a short recording period with the added benefit of inbuilt HRV analysis/reporting software offers several potential translational advantages for the HA environment over traditional HRV devices. These include better equipment portability, rapid data acquisition, simultaneous measurement of central blood pressure (BP^+^), early identification of fatigue and potentially HA-related illness. Munoz et al. previously examined the RMSSD [and normal-to-normal intervals (SDNN)] agreement between 10 s and 240–300 s recordings using continuous beat-to-beat pressure recordings on the middle finger pressure recording device (Portapres^®^ FMS Finapres Medical systems) (23). They observed a stronger correlation (*r* = 0.758–0.764) and agreement between the separate 10 s recordings and the 240–300 s recording than that observed in our study. HRV values for RMSSD were 1–2 ms higher with the longer vs. shorter recording. Importantly, in the study of Munoz et al. the 10 s measures were done in triplicate and in quiet temperature-controlled room (23). This could, in part explain, the stronger agreements and narrower limits of agreement between the ultra-short and standard HRV measures. In our study, whilst the 10 and 300 s were done sequentially they were conducted a four different altitudes (SL, 3,619, 4,600, and 5,140 m), temperatures, partial pressure of oxygen, and room environments (tent or hard structures).

Autonomic balance is under constant sympatho-parasympathetic flux that is highly dependent on the individual, HA environment and is subject to marked diurnal variability (9, 28). This might help to explain the inconsistencies in the data linking changes in HA to HRV and its relationship to AMS (7, 8, 28). These relationships are further complicated by the broad range of HRV time and frequency-domain HRV parameters available and sensors used to detect cardiac inter-beat intervals. In this study the early ascent profile was very gradual from 1,030 to 3,619 m over 7 days. This may be a reason for the higher RMSSD values, with both methods, at 3,619 m vs. SL baseline. In our study baseline HRV was measured approximately 6 weeks before the HA exposure. Consequently, it remains uncertain whether the higher HRV at 3,619 m vs. SL is due to early acclimatisation and improved fitness over the first week of ascent or a fall compared to baseline measures (if they had been taken at the actual start of the trek at HA) among participants who gained fitness over the previous 6 weeks preparation (after the SL baseline HRV measurement). In either case our data does convincingly demonstrate that above 3,619 m when the speed of severity of ascent intensifies that HRV does fall and is correlated with the degree of hypoxia.

This study has several limitations that should be acknowledged. The 10 s inter-beat interval acquired using the BP^+^ device was taken immediately before, and not during, the 300 s HRV recording period. This could be a source of bias as the participants would have been less rested. This protocol was done to fully compare the 10 s HRV with the 300 s gold standard HRV recording without the potential effects of blood pressure insufflation on HRV (33). Whilst the sampling frequencies of the BP^+^ and CMH were similar these devices use completely different fiducial points to determine the inter-beat interval with the BP^+^ device using the foot-to-foot interval of the brachial arterial wave whereas the CMH device uses the peak-to-peak R-wave of a single lead ECG. Hence, this was a comparison not only of recording duration but of detection site (upper arm vs. chest) and sensor method (arterial pressure waveform vs. single lead ECG) across multiple altitudes. The process from ECG R-wave generation (CMH device) to brachial arterial waveform detection (Uscom BP^+^) involves several transformation steps of physical properties from cardiac depolarisation to the ejection of blood into the arterial tree. Consequently, the individual influence of these factors cannot be determined and may lead to be systemic bias. The participant numbers were lower at the highest altitude which is not uncommon at very HA. This could be a source of potential bias as the non-participants at this altitude are more likely to have HA-related illness and lower HRV. Finally, the BP^+^ device does not provide user software to allow the manual exclusion or inclusion or over and under-sensed arterial waveforms and artefacts which is possible with the CMH system and is another potential source of bias.

## Conclusion

In conclusion, ultra-short 10 s HRV using a brachial cuff for signal acquisition are broadly consistent with that obtained usual a traditional 5-min gold standard HRV RMSSD measurement. However, their agreement is moderate. Increasing HA above 3,619 m is associated with a net fall in HRV. Neither method of HRV predicted AMS.

## Data Availability Statement

The raw data supporting the conclusions of this article will be made available by the authors, without undue reservation.

## Ethics Statement

The studies involving human participants were reviewed and approved by the Ministry of Defence Research and Ethics Committee. The patients/participants provided their written informed consent to participate in this study.

## Author Contributions

All authors have contributed to the design of the study and the writing of the manuscript. CB wrote the first draft of the manuscript and undertook all of the analyses. CB and AM went to Dhaulagiri to support the study.

## Conflict of Interest

The authors declare that the research was conducted in the absence of any commercial or financial relationships that could be construed as a potential conflict of interest.

## Publisher’s Note

All claims expressed in this article are solely those of the authors and do not necessarily represent those of their affiliated organizations, or those of the publisher, the editors and the reviewers. Any product that may be evaluated in this article, or claim that may be made by its manufacturer, is not guaranteed or endorsed by the publisher.
